# The role of voltage-gated calcium channels in neurotransmitter phenotype specification: Coexpression and functional analysis in *Xenopus laevis*

**DOI:** 10.1002/cne.23547

**Published:** 2014-01-01

**Authors:** Brittany B Lewis, Lauren E Miller, Wendy A Herbst, Margaret S Saha

**Affiliations:** Department of Biology, College of William and Mary,Williamsburg, Virginia, 23185

**Keywords:** calcium activity, glutamate, GABA, embryo, development

## Abstract

Calcium activity has been implicated in many neurodevelopmental events, including the specification of neurotransmitter phenotypes. Higher levels of calcium activity lead to an increased number of inhibitory neural phenotypes, whereas lower levels of calcium activity lead to excitatory neural phenotypes. Voltage-gated calcium channels (VGCCs) allow for rapid calcium entry and are expressed during early neural stages, making them likely regulators of activity-dependent neurotransmitter phenotype specification. To test this hypothesis, multiplex fluorescent in situ hybridization was used to characterize the coexpression of eight VGCC α1 subunits with the excitatory and inhibitory neural markers *xVGlut1* and *xVIAAT* in *Xenopus laevis* embryos. VGCC coexpression was higher with *xVGlut1* than *xVIAAT*, especially in the hindbrain, spinal cord, and cranial nerves. Calcium activity was also analyzed on a single-cell level, and spike frequency was correlated with the expression of VGCC α1 subunits in cell culture. Cells expressing *Ca_v_2.1* and *Ca_v_2.2* displayed increased calcium spiking compared with cells not expressing this marker. The VGCC antagonist diltiazem and agonist (−)BayK 8644 were used to manipulate calcium activity. Diltiazem exposure increased the number of glutamatergic cells and decreased the number of γ-aminobutyric acid (GABA)ergic cells, whereas (−)BayK 8644 exposure decreased the number of glutamatergic cells without having an effect on the number of GABAergic cells. Given that the expression and functional manipulation of VGCCs are correlated with neurotransmitter phenotype in some, but not all, experiments, VGCCs likely act in combination with a variety of other signaling factors to determine neuronal phenotype specification. J. Comp. Neurol. 522:2518–2531, 2014.

Changes in intracellular calcium concentration are implicated in a wide array of neurodevelopmental events ranging from neural induction to neurite outgrowth and synapse refinement (reviewed in Rosenberg and Spitzer, 2011; Leclerc et al., 2011). Although the mechanisms mediating spontaneous calcium activity during early neural development are not fully understood, it is known that voltage-gated calcium channels (VGCCs) play a role in regulating this activity. Essential for transducing changes in membrane potential into calcium activity that triggers cellular responses (reviewed in Barbado et al., 2009; Catterall, 2011; Turner et al., 2011), the family of 10 VGCC α1 subunits, which contain the pore-forming loop and determine the channel's physiological characteristics, is widely expressed in the developing nervous system (Lewis et al., 2009; Sanhueza et al., 2009; Morton et al., 2013). Moreover, calcium influx via VGCCs is known to regulate neural plate formation (Papanayotou et al., 2013), differentiation of neural progenitor cells (Lepski et al., 2013), dendrite morphogenesis (Nishiyama et al., 2011), axon outgrowth (Homma et al., 2006; Lu et al., 2009; Huang et al., 2012), and synaptic plasticity (Kasyanov et al., 2004; Takahashi and Magee, 2009).

A growing body of recent evidence suggests that calcium activity also plays an important and relatively novel role during neural development, namely, mediating neurotransmitter phenotype specification. The work of Spitzer and Borodinsky has shown that higher frequencies of calcium spiking lead to the specification of inhibitory phenotypes, whereas lower frequencies of calcium spiking result in the specification of excitatory phenotypes, a phenomenon that occurs both in vitro and in vivo (reviewed in Spitzer, 2012; Borodinsky et al., 2014). Subsequent work has elucidated the molecular mechanisms leading from calcium entry to the phenotype specification. Upregulating calcium activity via ion channel misexpression leads to the phosphorylation of cJun and subsequent repression of transcription factor Tlx3, which increases the number of γ-aminobutyric acid (GABA)ergic phenotypes and decreases the number of glutamatergic phenotypes; downregulating calcium activity produces the opposite result (Borodinsky et al., 2004; Marek et al., 2010). However, the molecular mechanisms governing the entry of calcium into prospective neurons remain unknown. Given the widespread expression of the VGCCs during neural plate and tube stages, these channels have been hypothesized to serve as candidates for mediating this activity-dependent neurotransmitter phenotype specification (Spitzer et al., 2002).

To begin to test the hypothesis that VGCCs mediate neurotransmitter phenotype choice, we have analyzed the coexpression of the VGCC α1 subunits with markers of glutamatergic (*xVGlut1*) and GABAergic or glycinergic (*xVIAAT*) neurotransmitter identity in *Xenopus laevis*, the species in which the role of calcium activity in neurotransmitter determination was first elucidated. Here we show that although there is no strict one-to-one colocalization between VGCCs and neurotransmitter phenotype, there are regions of significant colocalization. In addition, we correlated spiking behavior with VGCC expression in pharmacologically manipulated presumptive neurons to demonstrate that inhibiting or activating VGCCs alters neurotransmitter choice on a single-cell level. Taken together, these data suggest a role for these channels in mediating the activity associated with neurotransmitter phenotype specification.

## MATERIALS AND METHODS

### Animal use

Embryos were obtained by the natural mating of *Xenopus laevis* injected with human chorionic gonadotropin as described by Sive et al. (2000). Staging of embryos was performed according to Nieuwkoop and Faber (1994). Animal care and use protocols were performed in accordance with the regulations established by the Institutional Animal Care and Use Committee at the College of William and Mary.

### Whole-mount expression analysis

Antisense mRNA probes (Table1) were generated for eight of the VGCC α1 subunits, *Ca_v_1.2*, *Ca_v_1.2*, *Ca_v_1.3*, *Ca_v_1.4*, *Ca_v_2.1*, *Ca_v_2.3*, *Ca_v_3.1*, and *Ca_v_3.2*, and labeled with digoxigenin-11-UTP (Roche, Indianapolis, IN) as previously described (Lewis et al., 2009). Antisense mRNA probes for *xVGlut1* and *xVIAAT* were generated and labeled with fluorescein-12-UTP (Roche) (Gleason et al., 2003; Wester et al., 2008). Probes were synthesized in vitro by using standard techniques as described by Sambrook and Russell (2001). Multiplex fluorescence in situ histochemistry (FISH) analysis was performed on whole-mount *Xenopus laevis* early swimming tadpole stage embryos, using tyramide signal amplification to develop fluorescein and Cy3 fluorescence as described in Davidson and Keller (1999). For histological analysis, embryos were fixed in 1.6 M sucrose in phosphate-buffered saline for at least 12 hours at 4°C, embedded in tissue freezing medium (Triangle Biomedical Sciences, Durham, NC) at −20°C, cryosectioned into 18-μm transverse slices, and mounted onto slides for imaging using laser scanning confocal microscopy (Zeiss LSM 510). Histological sections were imaged at the 20× objective, with a zoom of 1× for brain and spinal cord images and a zoom of 1.2× for retinal images.

**Table 1:** Probe Sequences for In Situ Hybridization

Gene	Genbank accession no.	Bases
Ca_v_1.2	GQ120626	1–1,781
*Ca_v_1.3*	GQ120627	1–1,215
*Ca_v_1.4*	GQ120629	1–933
*Ca_v_2.1*	GQ120624	1–1,130
*Ca_v_2.2*	GQ120625	1–485
*Ca_v_2.3*	GQ120628	1–1,219
*Ca_v_3.1*	GQ120630	1–1,973
*Ca_v_3.2*	GQ120631	1–2,129
*xVGlut1*	AF548627	104–1,741
*xVIAAT*	NM_001086492	440–1,903
*xGAD67*	U38225	454–1,289

Antisense RNA probes were generated to hybridize to *Xenopus laevis* mRNA sequences for VGCC α1 subunits and neurotransmitter phenotype markers.

Images were taken by using the green fluorescein channel (excitation 488 nm, laser power 3.1%) and the red Cy3 channel (excitation 543 nm, laser power 14.9–16.9%). Detector gain and amplifier offset were adjusted for both channels to acquire an optimal signal. Detector gain was increased until regions showing signal saturated the photomultiplier tube and background regions did not. Amplifier offset was decreased until the intensity of background regions dropped just below the level of detection. “Coexpression” was defined as red and green signal present in the same cell, as indicated by yellow signal in the composite image.

### Primary cell culture

Neural tissue was dissected from stage 14, 18, and 22 *Xenopus laevis* embryos in modified Ringer's solution (MR) (Chang and Spitzer, 2009) supplemented with 1 mg/ml collagenase B (Roche) to facilitate dissections. After dissection, explants were transferred to a calcium- and magnesium-free (CMF) solution (Gu et al., 1994) and allowed to dissociate for 1 hour. Cells were plated on 35-mm Nunclon dishes (Cellattice; Nexcelom, Lawrence, MA) containing MR and were allowed to settle to the bottom of the plate for 1 hour. All steps of this procedure were performed at room temperature (22°C).

### Calcium imaging

For calcium imaging experiments, cells were incubated in 2.5 μM Fluo4-AM (Invitrogen Molecular Probes, Carlsbad, CA) with 0.01% Pluronic F-127 for 1 hour at room temperature. Cells were rinsed with MR in three successive washes. Two hours after they were initially plated, cells were imaged with confocal laser scanning microscopy (Zeiss LSM 510). Calcium imaging was recorded for 2 hours. The Argon 488-nm laser was set to 4% of its maximum 30 mW power, and the plate was scanned every 8 seconds for a total of 900 frames. Cells were fixed in 1X MEMFA for 30 minutes and dehydrated in 100% ethanol (Sive et al., 2000).

### Calcium activity analysis

Calcium activity was examined by using ImageJ (NIH). Stationary cells were circled manually to create regions of interest (ROIs). Average fluorescence intensity was examined in each of the 900 frames acquired during the calcium image and normalized to account for the gradual increase in baseline intensity seen in all cells due to gradual photo-bleaching, according to the equation: *F* = (*F*_R_ − *F*_B_)/(*F*_0_ − *F*_B_), where *F*_R_ is the raw fluorescence value within the ROI, *F*_B_ is background fluorescence, *F*_0_ is the average fluorescence of the past 10 frames, and *F* is the normalized value of the fluorescence intensity. This normalization process could result in an apparent undershoot if the raw fluorescence value of the current frame is below the average value of the previous 10 frames. Spikes were defined as a rise in fluorescence 50% above the baseline (0.5 units above the baseline of 1).

### Pharmacology

Neural tissue was dissected and dissociated in the same manner as calcium-imaged cultures, at the neural plate (st. 14), neural fold (st. 18), and neural tube (st. 22) stages. Cells were plated in MR containing 10 μM or 100 μM diltiazem (Sigma, St. Louis, MO), 1 μM or 10 μM (−)BayK-8644 (Sigma), MR alone, or MR with 0.05% dimethylsulfoxide (DMSO; BayK-8644 experiments only). The concentrations of diltiazem used in these experiments have previously been demonstrated to decrease calcium currents for VGCC α1 subunits in cell culture (Cai et al., 1997; De Paoli et al., 2002), and the concentrations of (−)BayK 8644 used were shown to increase calcium activity in amphibian explants and cell culture (Moreau et al., 1994; Takano et al., 2011). Cultures were fixed in 1X MEMFA when intact sibling embryos reached the swimming tadpole stage (35–36), and then stored in ethanol at −20°C.

### Expression analysis in primary cell culture

Antisense mRNA probes (Table1) were generated for *Ca_v_1.2*, *Ca_v_1.2*, *Ca_v_1.3*, *Ca_v_1.4*, *Ca_v_2.1*, *Ca_v_2.3*, *Ca_v_3.1*, *Ca_v_3.2* (Lewis et al., 2009), *xVGlut1* (Gleason et al., 2003), and *xGAD67* (Li et al., 2006) and used for expression analysis. Whereas *xVIAAT* was selected as a general inhibitory neural marker in whole-mount coexpression experiments, the specifically GABAergic probe *xGAD67* was selected for cell culture experiments because previous studies have demonstrated that calcium spike frequency regulates *xGAD67* expression in vitro (Watt et al., 2000). High-stringency FISH was performed on cell cultures using an anti-digoxigenin peroxidase antibody (Roche) and fluorescein–tyramide as the color substrate, following the protocol of Davidson and Keller (1999) with minor modifications as outlined by McDonough et al. (2012). Sense probes were used to determine background level of fluorescence.

### Statistical analysis of calcium-imaged plates

ROIs were divided into three categories: “positives,” those expressing the gene of interest, “negatives,” those not expressing the gene, and “unknowns,” cells that washed from the plate between calcium imaging and in situ hybridization analysis. The Mann–Whitney U-test was utilized to rank ROIs according to the number of spikes exhibited during the 2-hour image and to compare the activity in positive and negative ROIs, using programs written with MATLAB (MathWorks, Natick, MA). *P* values ≤0.05 were considered significant.

### Statistical analysis of pharmacologically exposed plates

The number of cells expressing the neurotransmitter phenotype marker of interest was counted, and the proportion of positive cells was compared among treatment and control plates using the two-sample z-test. *P* values ≤ 0.05 were considered significant.

## RESULTS

### Coexpression of VGCC α1 subunits with *xVGlut1*

**Figure 1:**
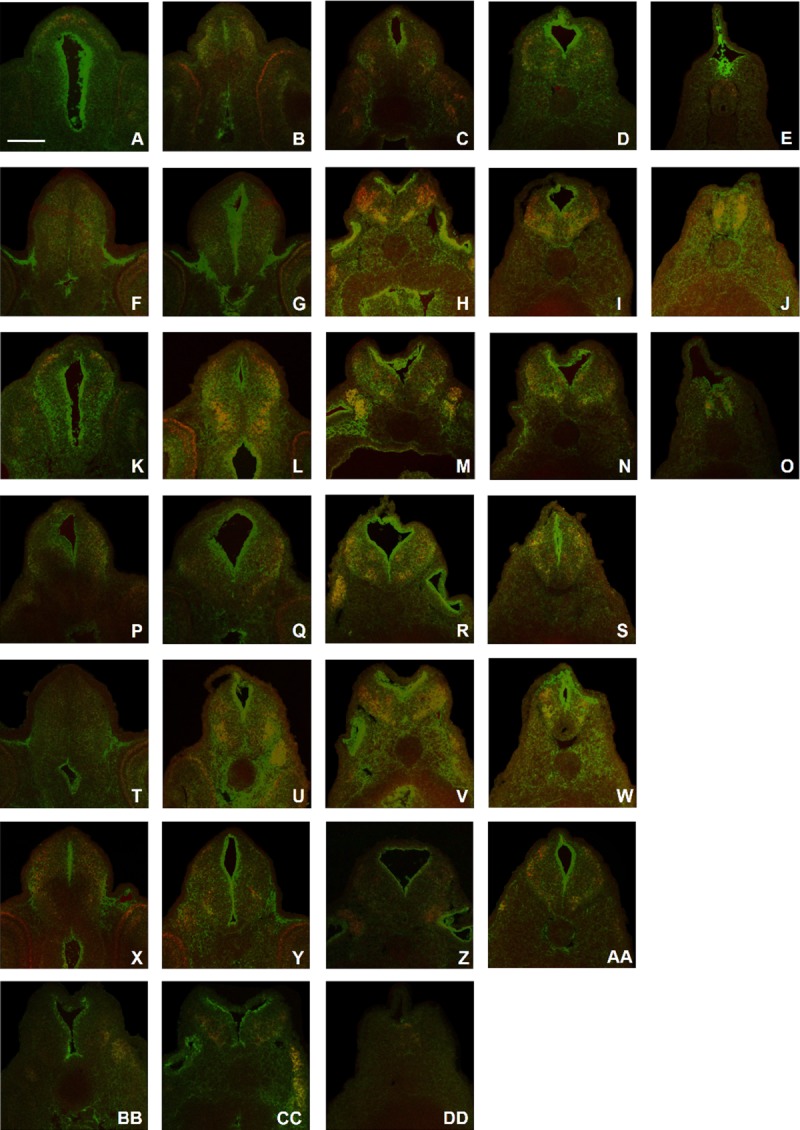
Coexpression patterns of *xVGlut1* and VGCC α1 subunits in the central nervous system of *Xenopus laevis* swimming tadpole embryos. VGCC subunit expression is labeled with fluorescein (green), and *xVGlut1* expression is labeled with Cy3 (red). Coexpression is indicated by the yellow overlap of both channels. A–E: *Ca_v_1.2* coexpression with *xVGlut1* in the (A) forebrain, (B) midbrain, (C) hindbrain, (D) anterior spinal cord, (E) and posterior spinal cord. F–J: *Ca_v_1.3* coexpression in the (F) forebrain, (G) midbrain, (H) hindbrain, (I) anterior spinal cord, (J) and posterior spinal cord. K–O: *Ca_v_2.1* coexpression in the (K) forebrain, (L) midbrain, (M) hindbrain, (N) anterior spinal cord, and (O) posterior spinal cord. P–S: *Ca_v_2.2* coexpression in the (P) forebrain, (Q) midbrain, (R) hindbrain, (S) spinal cord. T–AA: *Ca_v_3.1* coexpression in the (T) forebrain, (U) midbrain, (V) hindbrain, (W) spinal cord. *Ca_v_3.2* coexpression in the (X) forebrain, (Y) midbrain, (Z) hindbrain, and (AA) spinal cord. BB–DD: *Ca_v_2.3* coexpression in the (BB) midbrain, (CC) hindbrain, and (DD) spinal cord. For the assistance of color-blind readers, a magenta–green copy of this figure is provided as Supplementary Figure 1. Scale bar = 100 μm in A (applies to A–DD).

**Figure 2:**
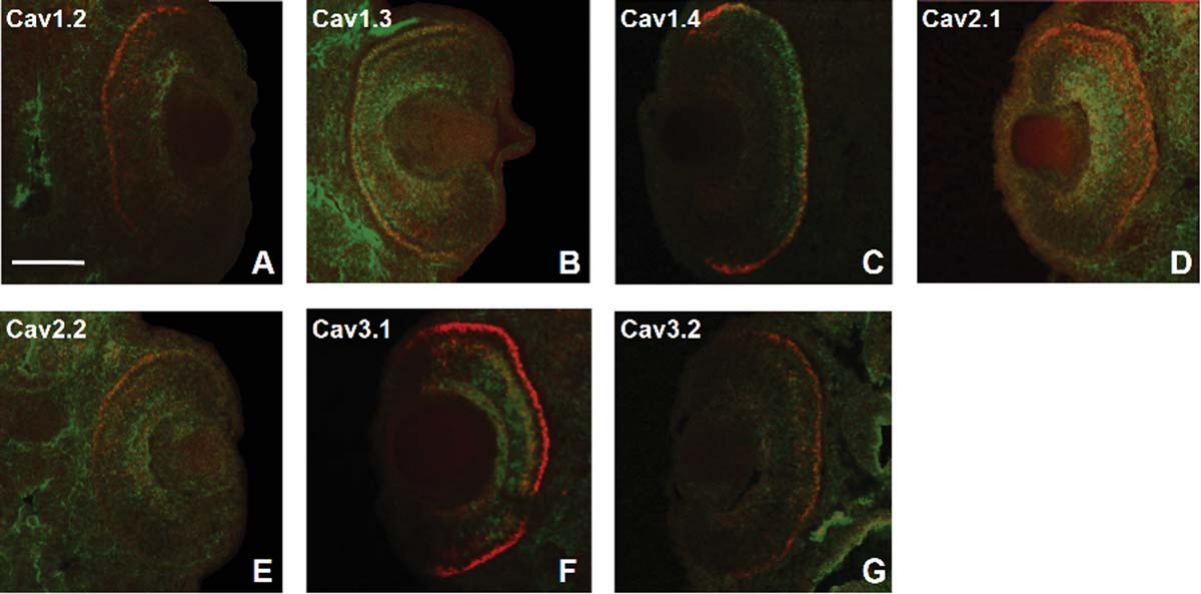
Coexpression patterns of *xVGlut1* and VGCC α1 subunits in the retina of *Xenopus laevis* swimming tadpole embryos. VGCC subunit expression is labeled with fluorescein (green), and *xVGlut1* expression is labeled with Cy3 (red). Coexpression is indicated by the yellow overlap of both channels. A–G: *xVGlut1* coexpression with (A) *Ca_v_1.2*, (B) *Ca_v_1.3*, (C) *Ca_v_1.4*, (D) *Ca_v_2.1*, (E) *Ca_v_2.2*, (F) *Ca_v_3.1*, and (G) *Ca_v_3.2*. For the assistance of color-blind readers, a magenta–green copy of this figure is provided as Supplementary Figure 2. Scale bar = 250 μm in A (applies to A–G).

To determine which, if any, VGCC α1 subunits are coexpressed with excitatory neurotransmitter markers, multiplex FISH analysis was performed, probing for VGCC markers and *xVGlut1*. VGCC α1 subunits are highly coexpressed with the glutamatergic marker throughout the developing nervous system, although many neurons expressing VGCCs do not express the neurotransmitter marker and vice versa (Table2). In the forebrain, *xVGlut1* is coexpressed with *Ca_v_1.2* in the dorsalmost tip (Fig. 1A), whereas overlap of *Ca_v_1.3* and *xVGlut1* occurs in a medial band extending from the dorsal end of the forebrain (Fig. 1F). *xVGlut1* coexpression also occurs along the lateral edge of the forebrain with *Ca_v_2.1* and *Ca_v_2.2* (Fig. 1K,P). Coexpression in the midbrain is highest between *xVGlut1* and *Ca_v_3.1* and is found in a ventral lateral region (Fig. 1U). *xVGlut1* coexpression is also found with *Ca_v_1.2* and *Ca_v_2.1* in the lateral midbrain (Fig. 1B,L). *xVGlut1* is coexpressed with *Ca_v_1.3* and *Ca_v_3.1* in the ventral hindbrain and in the spinal cord interneurons (Fig. 1H–J,V,W). Additional coexpression occurs with *Ca_v_2.2* along the lateral edge of the spinal cord (Fig. 1S). Very little coexpression is found in the retina, although *Ca_v_2.1* and *xVGlut1* do overlap in the ganglion cell layer (GCL) (Fig. 2D). In the cranial nerves, coexpression is found between *xVGlut1* and VGCCs in every cell in this region (Fig. 1G,M,R,V,W,Y,Z).

**Table 2:** Classification of coexpression patterns of VGCC α1 subunits and *xVGlut1*

	Forebrain	Midbrain	Hindbrain	Spinal Cord	Retina	Cranial Nerves
**% Cav1.2 cx**	+	++	+	+	-	-
**% vGlut cx**	++	++	+	++	-	-
**% Cav1.3 cx**	+	-	**+++**	**+++**	+	**+++**
**% vGlut cx**	++	-	**++**	**+++**	+	**+++**
**% Cav1.4 cx**	N/A	N/A	N/A	N/A	+	N/A
**% vGlut cx**	N/A	N/A	N/A	N/A	+	N/A
**% Cav2.1 cx**	+	++	+	+	+	**+++**
**% vGlut cx**	++	+++	+	++	++	**+++**
**% Cav2.2 cx**	+	+	++	+++	-	**+++**
**% vGlut cx**	+	++	++	+++	-	**+++**
**% Cav2.3 cx**	N/A	+	+	-	N/A	+
**% vGlut cx**	N/A	+	+	-	N/A	+
**% Cav3.1 cx**	-	**+**	**+++**	**+++**	+	**+++**
**% vGlut cx**	-	**+++**	**+++**	**+++**	+	**+++**
**% Cav3.2 cx**	-	+	+	++	+	**++**
**% vGlut cx**	-	+	+	++	+	**++**

For each coexpression (cx) experiment, the relative percentage of cells expressing both the VGCC subunit and *xVGlut1* (yellow FISH signal in Figs. 1–2) out of the total number of cells expressing the VGCC subunit (green and yellow FISH signal in Figs. 1–2) was determined. Additionally, the relative percentage of cells expressing both the VGCC subunit and *xVGlut1* (yellow FISH signal) out of the total number of cells expressing *xVGlut1* (red and yellow FISH signal) was determined. A single plus sign (+) indicates that <25% of the cells expressing the marker displayed coexpression. Two plus signs (++) indicates that 25–75% of the cells expressing the marker displayed coexpression, and three plus signs (+++) indicates >75% coexpression. Full coexpression between the two markers (>90%) is indicated in bold. Experiments without coexpression are marked with a minus sign (−).

### Coexpression of VGCC α1 subunits with *xVIAAT*

**Figure 3:**
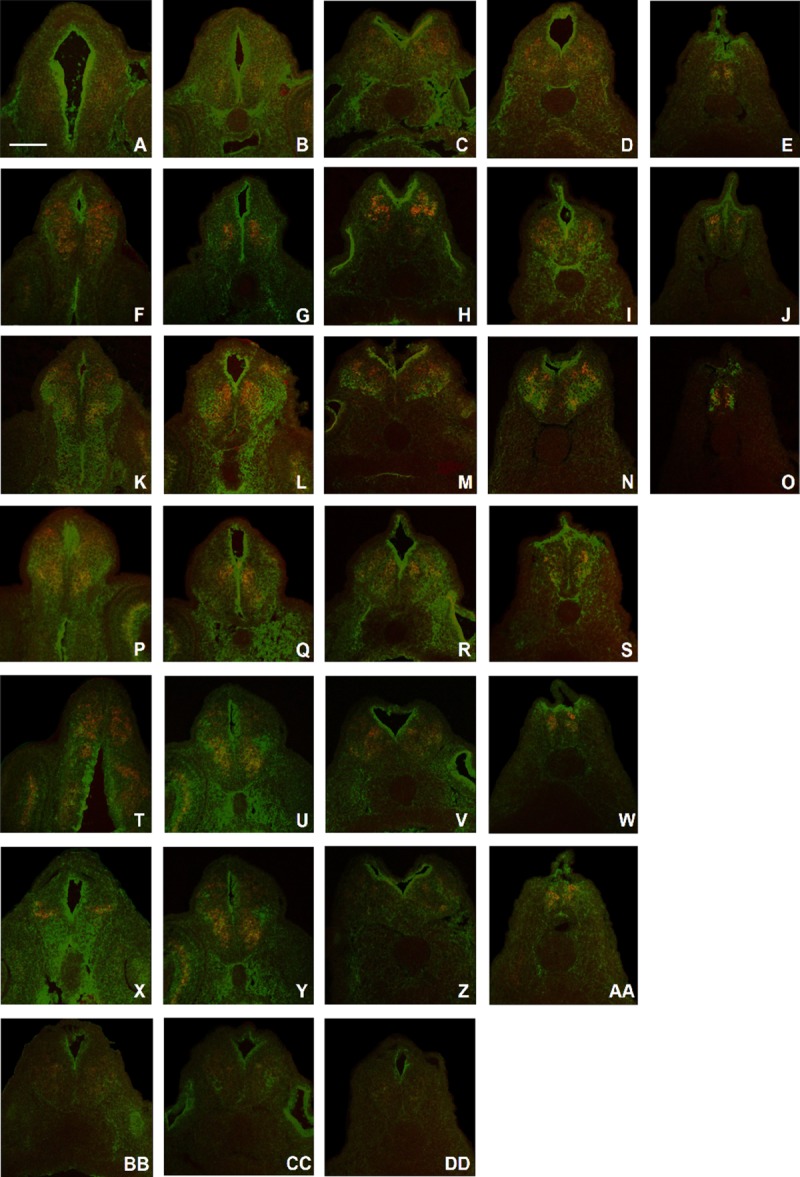
Coexpression patterns of *xVIAAT* and VGCC α1 subunits in the central nervous system of *Xenopus laevis* swimming tadpole embryos. VGCC subunit expression is labeled with fluorescein (green) and *xVIAAT* expression is labeled with Cy3 (red). Coexpression is indicated by the yellow overlap of both channels. A–D: *Ca_v_1.2* coexpression with *xVIAAT* in the (A) forebrain, (B) midbrain, (C) hindbrain, (D) anterior spinal cord, and (E) posterior spinal cord. F–J: *Ca_v_1.3* coexpression in the (F) forebrain, (G) midbrain, (H) hindbrain, (I) anterior spinal cord, and (J) posterior spinal cord. K–O: *Ca_v_2.1* coexpression in the (K) forebrain, (L) midbrain, (M) hindbrain, (N) anterior spinal cord, and (O) posterior spinal cord. P–S: *Ca_v_2.2* coexpression in the (P) forebrain, (Q) midbrain, (R) hindbrain, and (S) spinal cord. *Ca_v_3.1* coexpression in the (T) forebrain, (U) midbrain, (V) hindbrain, and (W) spinal cord. X–AA: *Ca_v_3.2* coexpression in the (X) forebrain, (Y) midbrain, (Z) hindbrain, and (AA) spinal cord. BB–DD: *Ca_v_2.3* coexpression in the (BB) midbrain, (CC) hindbrain, and (DD) spinal cord. For the assistance of color-blind readers, a magenta–green copy of this figure is provided as Supplementary Figure 4. Scale bar = 100 μm in A (applies to A–DD).

**Figure 4:**
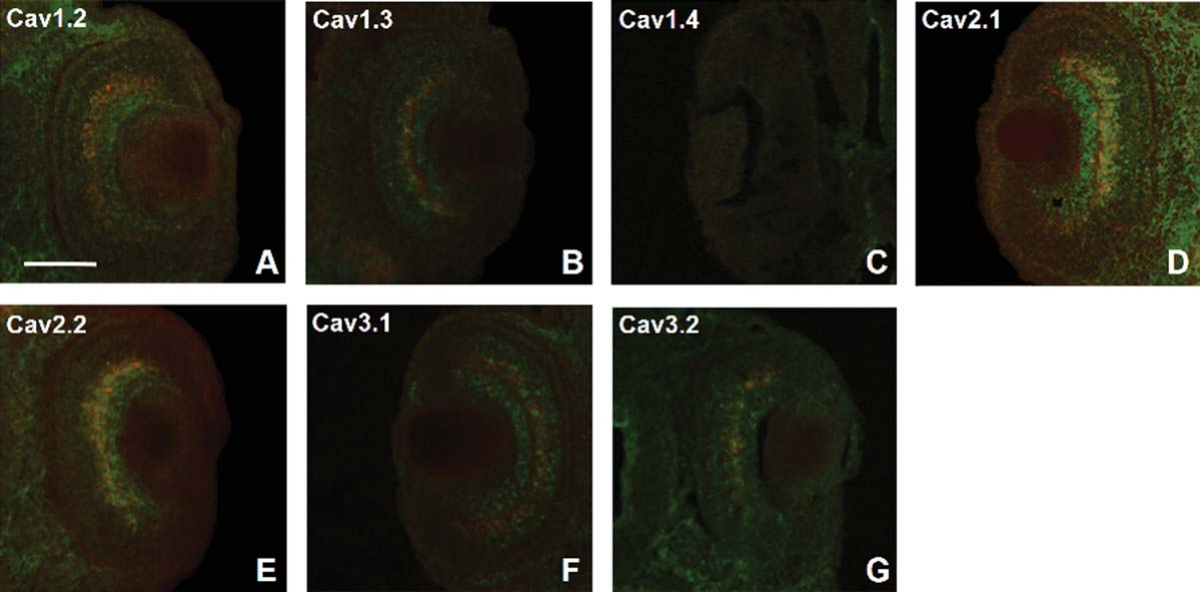
Coexpression patterns of *xVIAAT* and VGCC α1 subunits in the retina of *Xenopus laevis* swimming tadpole embryos. VGCC subunit expression is labeled with fluorescein (green), and *xVIAAT* expression is labeled with Cy3 (red). Coexpression is indicated by the yellow overlap of both channels. A–G: *xVIAAT* coexpression with (A) *Ca_v_1.2*, (B) *Ca_v_1.3*, (C) *Ca_v_1.4*, (D) *Ca_v_2.1*, (E) *Ca_v_2.2*, (F) *Ca_v_3.1*, and (G) *Ca_v_3.2*. For the assistance of color-blind readers, a magenta–green copy of this figure is provided as Supplementary Figure 3. Scale bar = 250 μm in A (applies to A–G).

To determine whether VGCC α1 subunits are coexpressed with inhibitory neurotransmitter markers, multiplex FISH analysis was performed, probing for VGCCs and *xVIAAT*. To a lesser extent, VGCC α1 subunits are coexpressed with the inhibitory neural marker (Table3). Forebrain and midbrain coexpression is restricted to ventral regions and occurs most prominently with *Ca_v_1.2*, *Ca_v_2.1*, and *Ca_v_2.2* (Fig. 3A,B,K,L,P,Q). Hindbrain coexpression is located dorsally and occurs with *Ca_v_1.2*, *Ca_v_1.3*, and *Ca_v_2.2* (Fig. 3C,H,R). In the spinal cord, coexpression is found with *Ca_v_1.2*, *Ca_v_1.3*, *Ca_v_2.1*, and *Ca_v_2.2* in the inhibitory commissural reciprocal interneurons (cINs) and ascending recurrent interneurons (aINs) (Fig. 3D,I,N,S). Retina coexpression occurs in the inner nuclear layer (INL) with *Ca_v_2.1* and *Ca_v_2.2* (Fig. 4D,E).

**Table 3:** Classification of coexpression patterns of VGCC α1 subunits and *xVIAAT*

	Forebrain	Midbrain	Hindbrain	Spinal Cord	Retina	Cranial Nerves
**% Cav1.2 cx**	++	+	+	+	+	N/A
**% VIAAT cx**	++	++	++	++	+	N/A
**% Cav1.3 cx**	+	+	++	+	-	N/A
**% VIAAT cx**	+	+	++	++	-	N/A
**% Cav1.4 cx**	N/A	N/A	N/A	N/A	+	N/A
**% VIAAT cx**	N/A	N/A	N/A	N/A	+	N/A
**% Cav2.1 cx**	+	+	-	+	++	N/A
**% VIAAT cx**	++	+++	+	++	+++	N/A
**% Cav2.2 cx**	++	+	+	+	++	N/A
**% VIAAT cx**	++	++	++	++	++	N/A
**% Cav2.3 cx**	N/A	+	+	+	N/A	N/A
**% VIAAT cx**	N/A	+	+	+	N/A	N/A
**% Cav3.1 cx**	-	+	+	+	+	N/A
**% VIAAT cx**	-	+	+	+	+	N/A
**% Cav3.2 cx**	+	+	+	+	+	N/A
**% VIAAT cx**	+	+	+	+	++	N/A

For each coexpression (cx) experiment, the relative percentage of cells expressing both the VGCC subunit and *xVIAAT* (yellow FISH signal in Figs. 4) out of the total number of cells expressing the VGCC subunit (green and yellow FISH signal in Figs. 4) was determined. Additionally, the relative percentage of cells expressing both the VGCC subunit and *xVIAAT* (yellow FISH signal) out of the total number of cells expressing *xVIAAT* (red and yellow FISH signal) was determined. A single plus sign (+) indicates that <25% of the cells expressing the marker displayed coexpression. Two plus signs (++) indicates that 25–75% of the cells expressing the marker displayed coexpression, and three plus signs (+++) indicates >75% coexpression. Experiments without coexpression are marked with a minus sign (−).

### *Ca_v_2.1* and *Ca_v_2.2* are correlated with high-frequency calcium activity at specific developmental stages

**Figure 5:**
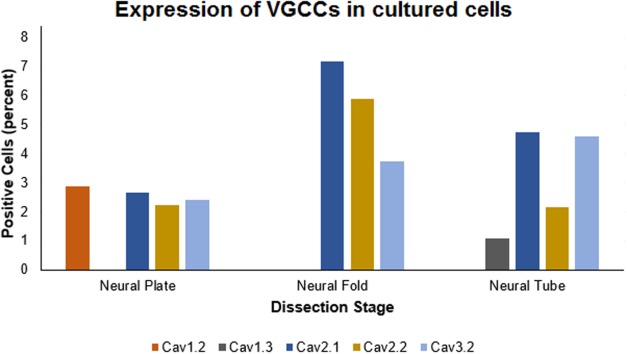
Expression of VGCCs in cultured cells. Expression of the five VGCC α1 subunits detected with FISH in neural cell cultures during early development. *Ca_v_1.2* and *Ca_v_1.3* are detected at neural plate and neural tube stages, respectively. *Ca_v_2.1*, *Ca_v_2.2*, and *Ca_v_3.2* are detected throughout the developmental period studied. *Ca_v_2.1* and *Ca_v_2.2* are expressed at highest levels at the neural fold stage, and *Ca_v_3.2* expression is highest at the neural tube stage. At the neural plate stage, *n* = 280 cells (*Ca_v_1.2*), 339 cells (*Ca_v_2.1*), 358 cells (*Ca_v_2.2*), and 578 cells (*Ca_v_3.2*). At the neural fold stage, *n* = 376 cells (*Ca_v_2.1*), 305 cells (*Ca_v_2.2*), and 403 cells (*Ca_v_3.2*). At the neural tube stage, *n* = 1,843 cells (*Ca_v_1.3*), 1,227 cells (*Ca_v_2.1*), 1,197 cells (*Ca_v_2.2*), and 631 cells (*Ca_v_3.2*).

After determining the coexpression of VGCC α1 subunits with neurotransmitter markers in whole-mount embryos, VGCC expression was correlated with calcium activity in primary cell culture. Expression was assessed at the neural plate (st. 14), neural fold (st. 18), and neural tube (st. 22) stages by using FISH. *Ca_v_1.2*, *Ca_v_2.1*, *Ca_v_2.2*, and *Ca_v_3.2* are the only channels detected in cultures dissected at the neural plate stage (Fig. 5). Whereas *Ca_v_2.1*, *Ca_v_2.2*, and *Ca_v_3.2* are subsequently expressed at neural fold and neural tube stage dissections, *Ca_v_1.2* is not detected in cells cultured after the neural plate stage. *Ca_v_1.3* is detected in cell cultures dissected at the neural tube stage.

**Figure 6:**
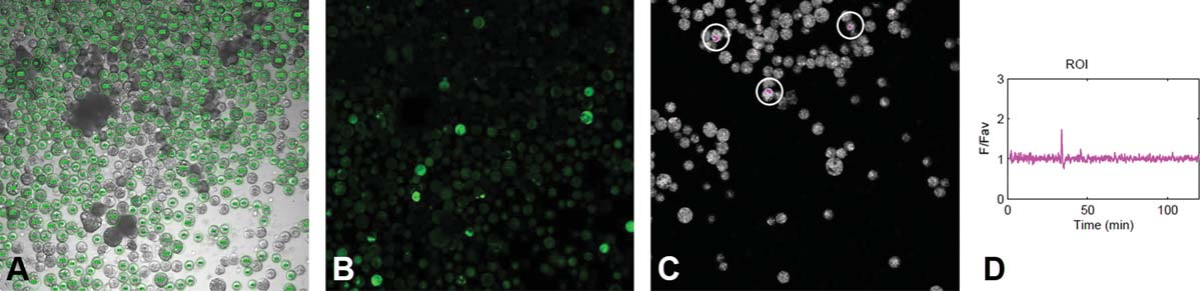
Sample image set from calcium imaging. A: Average brightfield image of cells during calcium imaging. B: Average Fluo4 fluorescence image of cells during calcium imaging. Bright regions indicate cells with high levels of calcium transients. C: Fluorescent image of cells after FISH. Positive cells are circled. D: Sample calcium activity data from a region of interest (ROI).

**Figure 7:**
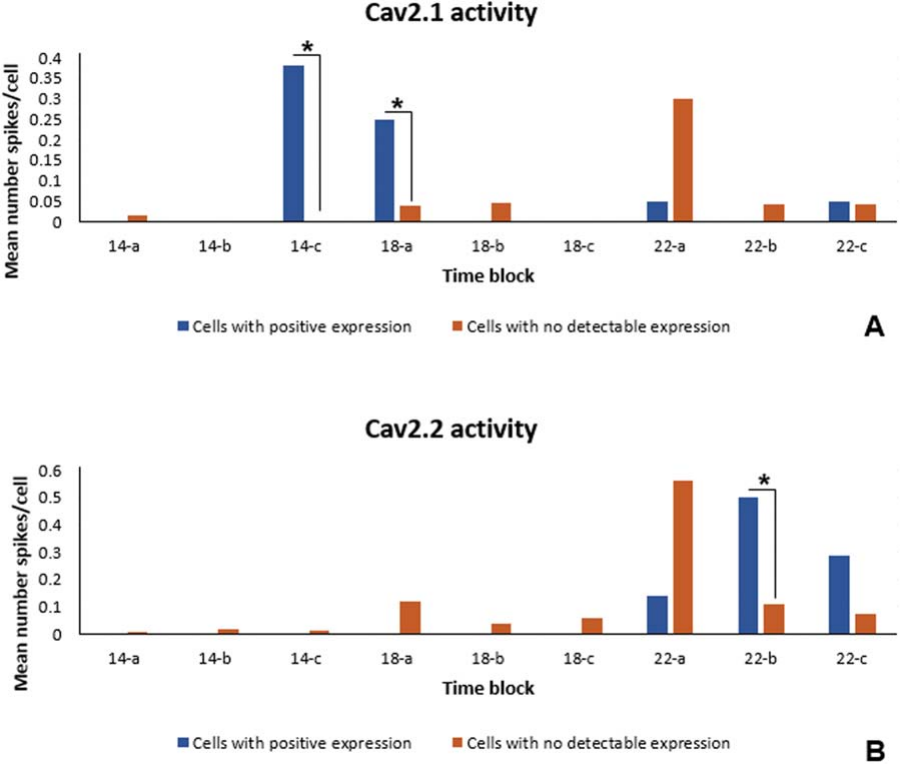
*Ca_v_2.1* and *Ca_v_2.2* activity. A,B: Comparison of calcium activity in cells exhibiting positive expression for *Ca_v_2.1* (A) and *Ca_v_2.2* (B) with cells displaying no detectable expression for this VGCC (negative cells). Activity for the cells was analyzed over a 2-hour imaging session, and each dissection (st.14, 18, or 22) was divided into three time blocks. A single time block represents 40 minutes of the image, i.e., “a” is *t* = 0 to *t* = 40 minutes, “b” is *t* = 40 minutes to *t* = 80 minutes, and “c” is *t* = 80 minutes to *t* = 120 minutes. Numbers of spikes in positive and negative cells were compared using the Mann–Whitney U-test. Comparisons with *P* values ≤ 0.05 are marked with an asterisk (*) on the figure. For *Ca_v_2.1* experiments, *n* = 340 cells (neural plate), 294 cells (neural fold), and 1,228 cells (neural tube). For *Ca_v_2.2* experiments, *n* = 357 cells (neural plate), 139 cells (neural fold), and 1,197 cells (neural tube).

As the only VGCC α1 subunits expressed in cultured cells dissected at neural plate, neural fold, and neural tube stages, *Ca_v_1.2*, *Ca_v_1.3*, *Ca_v_2.1*, *Ca_v_2.2*, and *Ca_v_3.2* were examined further in calcium activity imaging experiments (Fig. 6). Two-hour calcium images were performed on neuronal cell culture, and spiking data were analyzed in three 40-minute time blocks. The number of calcium spikes in cells with VGCC expression (positive cells) was compared with the number of spikes in cells without detectable expression (negative cells). *Ca_v_2.1* is correlated with high-frequency calcium activity in cell cultures dissected at the neural plate and neural fold stages (Fig. 7A) and *Ca_v_*2*2*.2*2* is correlated with high-frequency calcium activity in neural tube cultures (Fig. 7B). The *Ca_v_1.2*-, *Ca_v_1.3*-, and *Ca_v_3.2*-positive cells have equal spiking activity compared with cells negative for these markers.

### Pharmacological disruption of VGCC activity leads to changes in neurotransmitter phenotype specification

**Figure 8:**
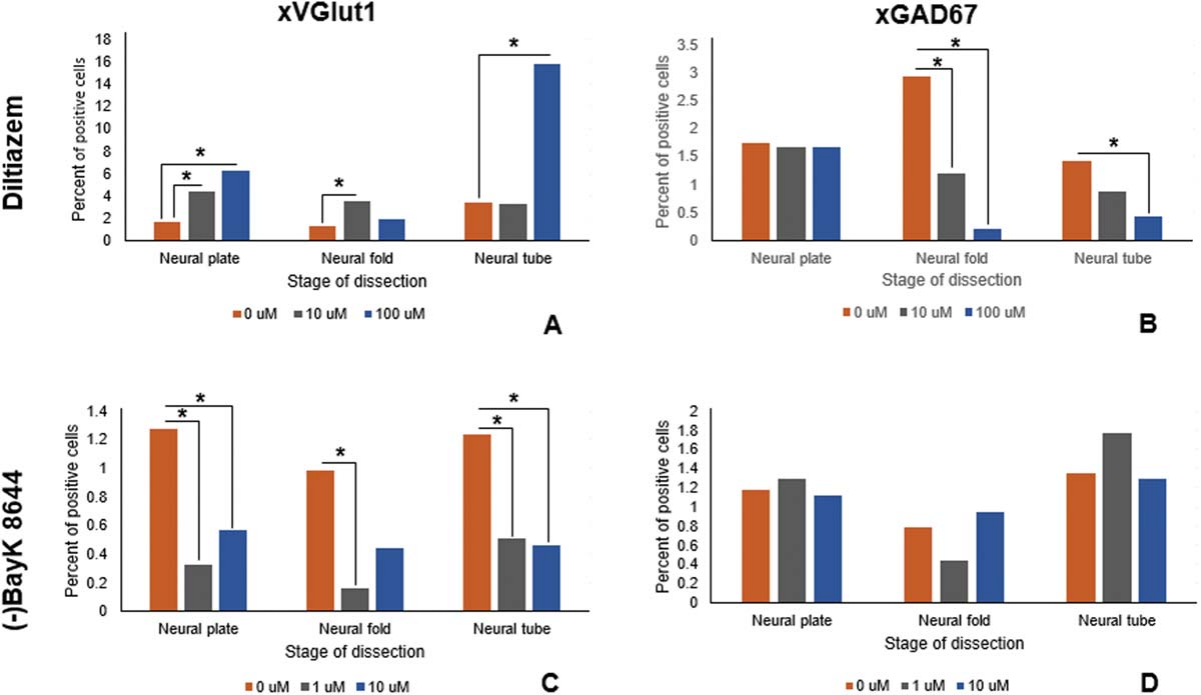
Pharmacological disruption of VGCC activity and neurotransmitter phenotype. Embryos were dissected at neural plate, neural fold, and neural tube stages, incubated in either a VGCC antagonist (diltiazem) or a VGCC agonist (BayK 8644), and then assayed for expression of the glutamatergic marker *xVGlut1* or the GABAergic marker *xGAD67*. The number of cells expressing the gene of interest (termed “positives”) and the number of cells not expressing the gene of interest (termed “negatives”) for representative fields of view were recorded. A two-sample z-test was used to compare the percent positive between treatment groups and controls. *P* ≤ 0.05 was recorded as significant. A: *xVGlut1* expression of diltiazem-exposed cells. B: *xGAD67* expression of diltiazem-exposed cells. C: *xVGlut1* expression of BayK 8644-exposed cells. D: *xGAD67* expression of BayK 8644-exposed cells. For diltiazem exposures with *xVGlut1* expression, *n* = 1,398 cells (0 μM, neural plate), 1,069 cells (10 μM, neural plate), 936 cells (100 μM, neural plate), 909 cells (0 μM, neural fold), 1,003 cells (10 μM, neural fold), 1,160 cells (100 μM, neural fold), 2,419 cells (0 μM, neural tube), 1,147 cells (10 μM, neural tube), and 160 cells (100 μM, neural tube). For diltizem exposures with *xGAD67* expression, *n* = 1,259 cells (0 μM, neural plate), 843 cells (10 μM, neural plate), 658 cells (100 μM, neural plate), 576 cells (0 μM, neural fold), 1,523 cells (10 μM, neural fold), 1,441 cells (100 μM, neural fold), 1,486 cells (0 μM, neural tube), 1,509 cells (10 μM, neural tube), and 1,171 cells (100 μM, neural tube). For BayK 8644 exposures with *xVGlut1* expression, *n* = 1,178 cells (0 μM, neural plate), 1,817 cells (10 μM, neural plate), 1,755 cells (100 μM, neural plate), 1,222 cells (0 μM, neural fold), 1,249 cells (10 μM, neural fold), 1,126 cells (100 μM, neural fold), 1,455 cells (0 μM, neural tube), 1,384 cells (10 μM, neural tube), and 1,519 cells (100 μM, neural tube). For BayK 8644 exposures with *xGAD67* expression, *n* = 851 cells (0 μM, neural plate), 1,777 cells (10 μM, neural plate), 1,075 cells (100 μM, neural plate), 1,018 cells (0 μM, neural fold), 674 cells (10 μM, neural fold), 745 cells (100 μM, neural fold), 1,560 cells (0 μM, neural tube), 1,469 cells (10 μM, neural tube), and 1,001 cells (100 μM, neural tube).

Because VGCCs are expressed in neural tissue during early development and certain VGCCs are correlated with specific spike frequencies in cell culture, the effect of the VGCC blocker diltiazem and agonist (−)Bayk 8644 on neurotransmitter phenotype specification was examined next. The percentage of cells expressing *xVGlut1* or *xGAD67* was assessed among treatment conditions, and a two-sample z-test was used to compare the percentage of positive cells in cultures exposed to diltiazem or (−)BayK 8644 with untreated controls. Exposure to the VGCC antagonist leads to an increase in glutamatergic neurons and a decrease in GABAergic neurons (Fig. 8A,B). Exposure to the VGCC agonist significantly decreases the percentage of cells expressing glutamatergic cells while having no effect on the number of GABAergic cells. (Fig. 8C,D)

## DISCUSSION

### Coexpression of VGCC α1 subunits with neurotransmitter phenotype markers

The goal of this study was to test the hypothesis that calcium signaling through VGCCs, at least in part, modulates neurotransmitter phenotype specification in developing *Xenopus laevis* embryos. An obvious prediction of this hypothesis is a clear pattern of colocalization of specific VGCCs with markers of neurotransmitter phenotype, at least on a regional level. Although coexpression experiments demonstrated that no VGCC α1 subunit has a simple one-to-one pattern of colocalization with either *xVGlut1* or *xVIAAT*, in most regions examined, VGCCs are coexpressed with *xVGlut1* or *xVIAAT* in some neurons, but VGCC expression is also found in neurons not expressing either of these markers. Conversely, *xVGlut1* and *xVIAAT* are found in neurons not expressing VGCC α1 subunits. However, several VGCC α1 subunits display preferential colocalization with either *xVGlut1* or *xVIAAT*. *Ca_v_1.3* is coexpressed more frequently with *xVGlut1* whereas *Ca_v_1.2* and *Ca_v_3.1* are coexpressed more frequently with *xVIAAT*. This differential expression suggests that specific VGCC subunits could mediate the determination of excitatory and inhibitory neurotransmitter phenotypes. Although *Ca_v_2.1* and *Ca_v_2.2* both display tight coexpression patterns, they are correlated with both *xVGlut1* and *xVIAAT* depending on the specific region (Tables 1, 2).

A caveat to this analysis is that VGCC α1 subunits display extensive alternative splicing (Rajapaksha et al., 2008; Zhang et al., 2010; Gardezi et al., 2010; Tuluc and Flucher, 2011; Tan et al., 2012). These variants have different functional characteristics, which can alter the sensitivity of depolarization-dependent calcium signaling across development and in different regions of the nervous system (reviewed in Lipscombe et al., 2013). It is possible that specific splice isoforms of VGCCs are important in the determination of particular neurotransmitter phenotypes. Because the probes used in these experiments may bind multiple splice isoforms of each VGCC α1 subunit, the patterns observed in colocalization experiments could include several individual patterns. Similarly, because *xVIAAT* marks both GABAergic and glycinergic phenotypes, there may be patterns of colocalization between particular VGCCs and either of these two phenotypes that cannot be distinguished in these experiments.

It is also important to note that the process of neurotransmitter phenotype specification likely occurs at earlier stages of *Xenopus* development than the stage examined in this study (early swimming tadpole). However, the expression patterns of VGCCs and neurotransmitter markers examined in this study are spatially consistent across *Xenopus* embryonic development (Gleason et al., 2003; Wester et al., 2008; Lewis et al., 2009), and coexpression patterns at later stages are indicative of coexpression at earlier stages. The swimming tadpole stage was selected for analysis because of the robust expression of VGCCs and neural markers. Additionally, coexpression of VGCCs and neurotransmitter markers may continue at these later stages, to maintain the appropriate neural phenotype. Neurotransmitter identity is not a stable fate; even after synapses have formed, changes in electrical activity can respecify the neurotransmitters expressed in a given neuron (reviewed in Spitzer, 2012). Neurotransmitter respecification is known to occur in the ventral suprachiasmic nucleus (vSCN), where dopaminergic neurons are recruited in response to increased sensory input (Dulcis and Spitzer, 2008). In the mossy fiber projection of the hippocampus in the adult brain, exclusively glutamatergic transmission is respecified to simultaneous glutamatergic and GABAergic signaling, in response to physiological stimulation of the dentate gyrus (Gutiérrez, 2002). Maintaining the appropriate neurotransmitter phenotype is ongoing, and the mechanisms that regulate this process, including electrical activity via VGCCs, are likely to persist throughout development.

### VGCC expression as a marker for neuronal subtypes

Although there was not a robust correlation between VGCC expression and neurotransmitter phenotype, the detailed characterization of these neural genes throughout the developing vertebrate nervous system can be used to identify subpopulations of neurons. Whereas the spatial expression of excitatory and inhibitory neurotransmitter phenotypes has been characterized during early embryogenesis throughout the developing brain (Gleason et al., 2003; Wester et al., 2008), spinal cord (reviewed in Roberts et al., 2012), and retina (Dullin et al., 2007), the specific VGCCs expressed in neural subpopulations is less well known. Several studies have characterized the expression of VGCCs during embryogenesis (Zhou et al., 2008; Lewis et al., 2009; Sanhueza et al., 2009), but this is the first comprehensive study to describe the expression of calcium channels and neural phenotype markers together. Coexpression patterns demonstrate which VGCCs are expressed in specific inhibitory and excitatory neural subpopulations.

Histological analysis revealed that, in many instances, VGCCs are expressed in discrete, highly specific regions of the nervous system. This distinct patterning of VGCCs may contribute to a diverse array of physiological properties in these neural subpopulations. The expression of *Ca_v_1.2* in the inhibitory subpopulations of the dorsal spinal cord (cINs and aINs) is of note, because this VGCC is strongly implicated in regulating neural gene expression and plasticity by activation of CREB (Dolmetsch et al., 2001; Wheeler et al., 2008). The glutamatergic descending interneurons (dINs) in the ventral spinal cord were found to express two developmentally important VGCCs: *Ca_v_1.3*, which contributes to calcium oscillations in pacemaker neurons (Guzman et al., 2009; reviewed in Vandael et al., 2013), and *Ca_v_2.1*, which mediates neurotransmitter release and is involved in synaptic competition (Hashimoto et al., 2011) and plasticity (Mochida et al., 2008; reviewed in Catterall et al., 2013). Knockout models have further demonstrated the neurodevelopmental importance of these VGCCs; *Ca_v_2.1* knockout mice display developmental abnormalities in in the cerebellum, including axonal swelling of Purkinje cells and a deficit in external granule cell layer (Jun et al., 1999), and *Ca_v_1.3* knockout mice have underdeveloped auditory brainstems with significantly fewer neurons in the lateral superior olive (Hirtz et al., 2011). Additionally, *Ca_v_1.3* knockout mice display elevated levels of glutamate, GABA, and serotonin, suggesting that VGCCs are necessary for the normal expression of neurotransmitters (Sagala et al., 2012).

### Correlation of calcium activity, VGCC expression, and neurotransmitter phenotype in cell culture

Although previous research shows that global manipulation of calcium activity leads to changes in the proportion of GABAergic and glutamatergic neurons in *Xenopus* embryos, no studies to date have investigated the relationships among calcium activity, VGCC expression, and neurotransmitter phenotype at a single-cell level. This study demonstrates that, of the five VGCC α1 subunits detected in cell cultures, expression of *Ca_v_2.1* and *Ca_v_2.2* is correlated most strongly with high frequencies of calcium activity. Specifically, cells cultured at the neural plate and neural fold stages that express *Ca_v_2.1* have higher spike counts during the 2-hour image than cells with no detectable expression and, at the neural tube stage, cultured cells that express *Ca_v_2.2* have a significantly higher spike frequency than cells with no expression for this VGCC. Interestingly, these two VGCC subunits display the highest coexpression with both inhibitory and excitatory neural markers.

The other three VGCCs with positive expression in culture, *Ca_v_1.2*, *Ca_v_1.3*, and *Ca_v_3.2*, are not associated with increased calcium activity in the time period examined, despite the coexpression patterns implying that *Ca_v_1.2* and *Ca_v_1.3* play a role in neurotransmitter phenotype specification, given their preferential coexpression with *xVIAAT* and *xVGlut1*, respectively. Because some VGCC α1 subunits are correlated with specific patterns of calcium activity, the effect of VGCC antagonist application on cultured cells was examined next. Cultured cells exposed to diltiazem express significantly more *xVGlut1*-positive cells and significantly fewer *xGAD67*-positive cells than untreated controls. The upregulation of the excitatory neurotransmitter marker *xVGlut1* in response to decreased calcium activity agrees with the result found by Borodinsky et al. (2004): injection of potassium channel mRNA to hyperpolarize cell membranes and decrease activity resulted in greater immunoreactivity to the excitatory transmitters glutamate and acetylcholine. The concomitant decrease in *xGAD67* mRNA expression seen in response to diltiazem exposure also supports the homeostatic model of neurotransmitter phenotype specification (Spitzer et al., 2005). Cultured cells exposed to (−)BayK 8644 express significantly fewer *xVGlut1* cells than control cultures at the neural plate, neural fold, and neural tube stages. However, the percentage of *xGAD67*-positive cells is similar in (−)BayK 8644 and control cultures, which suggests that other signaling factors contribute to neurotransmitter phenotype specification and, in certain cases, prevail over the effects of VGCC activation.

Many other receptors and ion channels are known to regulate early calcium signaling and are therefore likely candidates for mediating calcium-dependent neurotransmitter phenotype specification. Purinergic receptors, which include the ionotropic P2X family and the metabotropic P2Y family, are expressed as early as gastrula and neurula stages of development (reviewed in Massé and Dale, 2012). ATP signaling via purinergic receptors can induce calcium transients that are implicated in neural differentiation (reviewed in Glaser et al., 2013). It has been proposed that transient receptor potential cation channels (TRPCs), which allow calcium and sodium ions into the cell, may mediate calcium activity during development (reviewed in Leclerc et al., 2011). TRPCs are expressed as early as the blastula stages of *Xenopus laevis* and have been implicated in the production of calcium transients induced by fibroblast growth factor (FGF) signaling (Lee et al., 2009). TRPCs are involved in neurodevelopmental events including the guidance of *Xenopus* neuronal growth cones (Shim et al., 2005; Kerstein et al., 2013) and evoking calcium transients during rat neural stem cell proliferation (Fiorio Pla et al., 2005). These alternate mechanisms could explain why only a few VGCC α1 subunits were correlated with activity, why functional manipulation of VGCCs did not always alter neurotransmitter phenotype, and, in the aforementioned coexpression experiments, why neuronal phenotype markers were found in many regions in the absence of VGCCs. Further study will be needed to characterize the role of other calcium-related channels and receptors in activity-dependent neurotransmitter phenotype specification.

## ROLE OF AUTHORS

All authors had full access to all the data in the study and take responsibility for the integrity of the data and the accuracy of the data analysis. Study concept and design: MS Saha. Acquisition of data: LE Miller, BB Lewis. Analysis and interpretation of data: LE Miller, BB Lewis, WA Herbst. Drafting of the manuscript: WA Herbst. Critical revision of the manuscript for important intellectual content: MS Saha. Obtained funding: MS Saha. Administrative, technical, and material support: MS Saha. Study supervision: MS Saha.
