# Supplementary material for: The role of voltage-gated calcium channels in neurotransmitter phenotype specification: Coexpression and functional analysis in Xenopus laevis
Source: J Comp Neurol. 2014 Jan 1;522(11):2518–31. doi: 10.1002/cne.23547 (PMC4043876; doi:10.1002/cne.23547)
Supplement: Supplementary file 1 [file cne0522-2518-SD1.pdf]

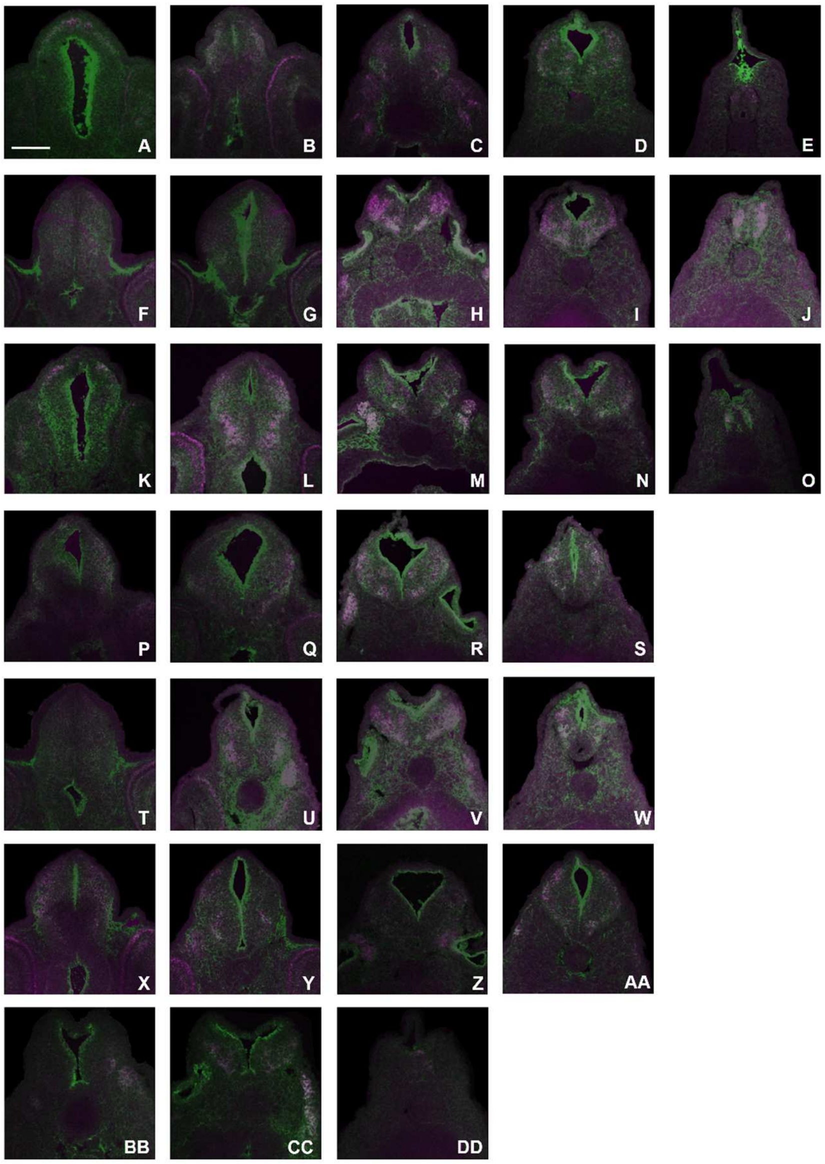

Supplementary Figure 1. (Magenta-green version of Figure 1 for the assistance of color-blind readers.) Coexpression patterns of xVGlut1 and VGCC α1 subunits in the central nervous system of Xenopus laevis swimming tadpole embryos. VGCC subunit expression is labeled with fluorescein (green) and xVGlut1 expression is labeled with Cy3 (red). Coexpression is indicated by the yellow overlap of both channels. Scale bar represents 100 μm. Cav1.2 coexpression with xVGlut1 in the (A) forebrain, (B) midbrain, (C) hindbrain, (D) anterior spinal cord, (E) posterior spinal cord. Cav¬1.3 coexpression in the (F) forebrain, (G) midbrain, (H) hindbrain, (I) anterior spinal cord, (J) posterior spinal cord. Cav2.1 coexpression in the (K) forebrain, (L) midbrain, (M) hindbrain, (N) anterior spinal cord, (O) posterior spinal cord. Cav2.2 coexpression in the (P) forebrain, (Q) midbrain, (R) hindbrain, (S) spinal cord. Cav3.1 coexpression in the (T) forebrain, (U) midbrain, (V) hindbrain, (W) spinal cord. Cav3.2 coexpression in the (X) forebrain, (Y) midbrain, (Z) hindbrain, (AA) spinal cord. Cav2.3 coexpression in the (BB) midbrain, (CC) hindbrain, (DD) spinal cord.
295x420mm (96 x 96 DPI)

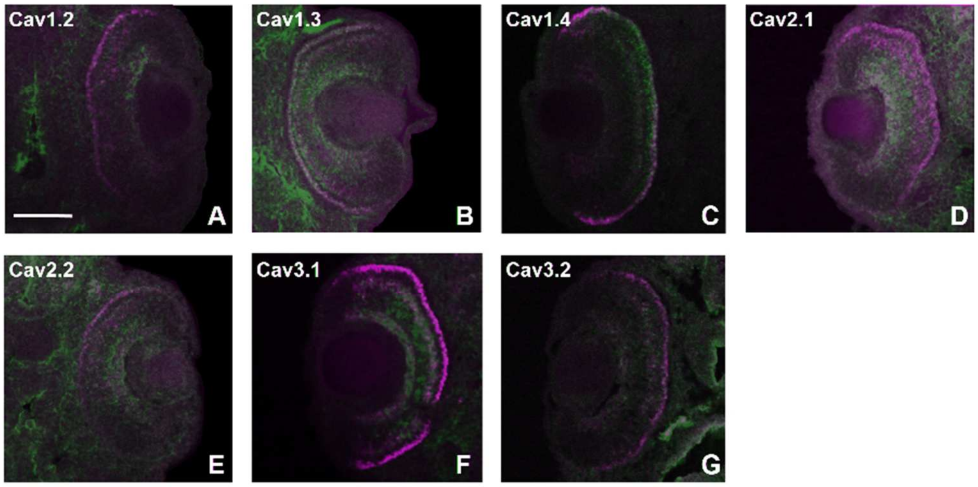

Supplementary Figure 2. (Magenta-green version of Figure 2 for the assistance of color-blind readers.) Coexpression patterns of xVGlut1 and VGCC α1 subunits in the retina of Xenopus laevis swimming tadpole embryos. VGCC subunit expression is labeled with fluorescein (green) and xVGlut1 expression is labeled with Cy3 (red). Coexpression is indicated by the yellow overlap of both channels. Scale bar represents 250 μm. xVGlut1 coexpression with (A) Cav1.2, (B) Cav1.3, (C) Cav1.4, (D) Cav2.1, (E) Cav2.2, (F) Cav3.1, (G) Cav3.2. A magenta-green copy of this figure is provided as Supplementary Figure 2.
244x124mm (96 x 96 DPI)

Review

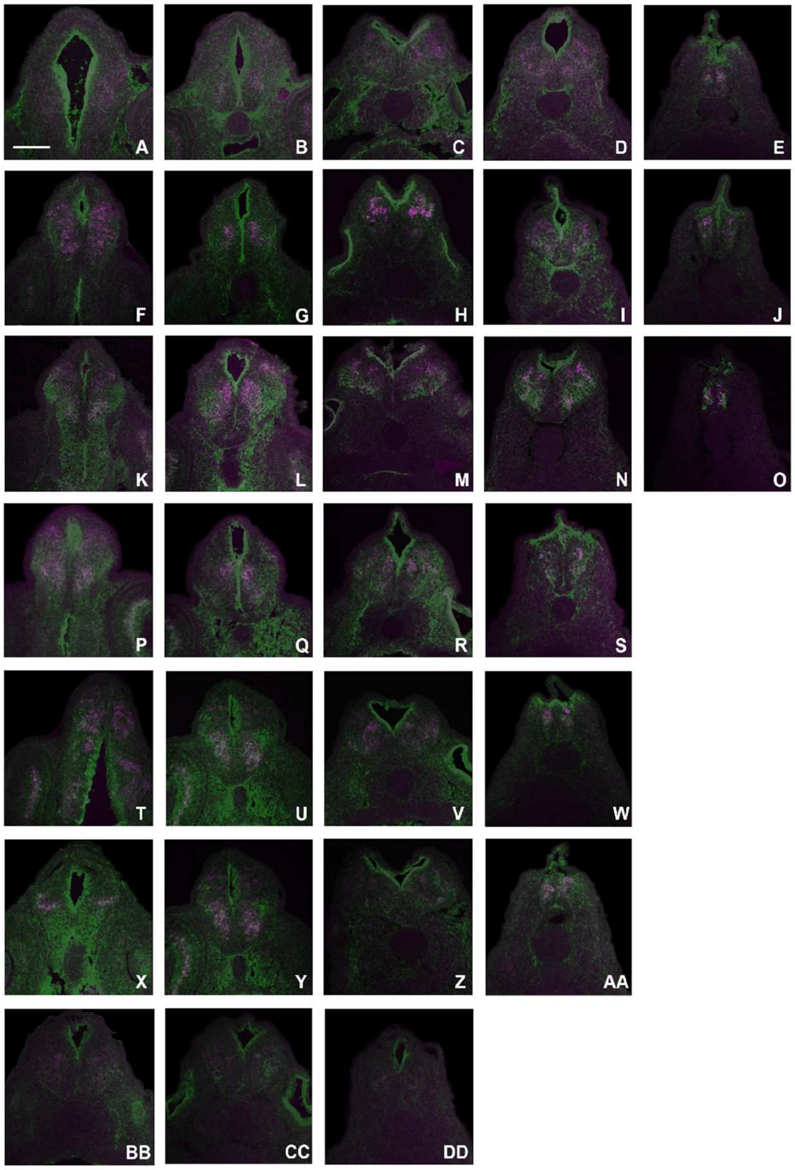

Supplementary Figure 3. (Magenta-green version of Figure 3 for the assistance of color-blind readers.) Coexpression patterns of xVIAAT and VGCC α1 subunits in the central nervous system of Xenopus laevis swimming tadpole embryos. VGCC subunit expression is labeled with fluorescein (green) and xVIAAT expression is labeled with Cy3 (red). Coexpression is indicated by the yellow overlap of both channels. Scale bar represents 100 µm. Cav1.2 coexpression with xVIAAT in the (A) forebrain, (B) midbrain, (C) hindbrain, (D) anterior spinal cord, (E) posterior spinal cord. Cav¬1.3 coexpression in the (F) forebrain, (G) midbrain, (H) hindbrain, (I) anterior spinal cord, (J) posterior spinal cord. Cav2.1 coexpression in the (K) forebrain, (L) midbrain, (M) hindbrain, (N) anterior spinal cord, (O) posterior spinal cord. Cav2.2 coexpression in the (P) forebrain, (Q) midbrain, (R) hindbrain, (S) spinal cord. Cav3.1 coexpression in the (T) forebrain, (U) midbrain, (V) hindbrain, (W) spinal cord. Cav3.2 coexpression in the (X) forebrain, (Y) midbrain, (Z) hindbrain, (AA) spinal cord. Cav2.3 coexpression in the (BB) midbrain, (CC) hindbrain, (DD) spinal cord. A magenta-green copy of this figure is provided as Supplementary Figure 3.
286x412mm (96 x 96 DPI)

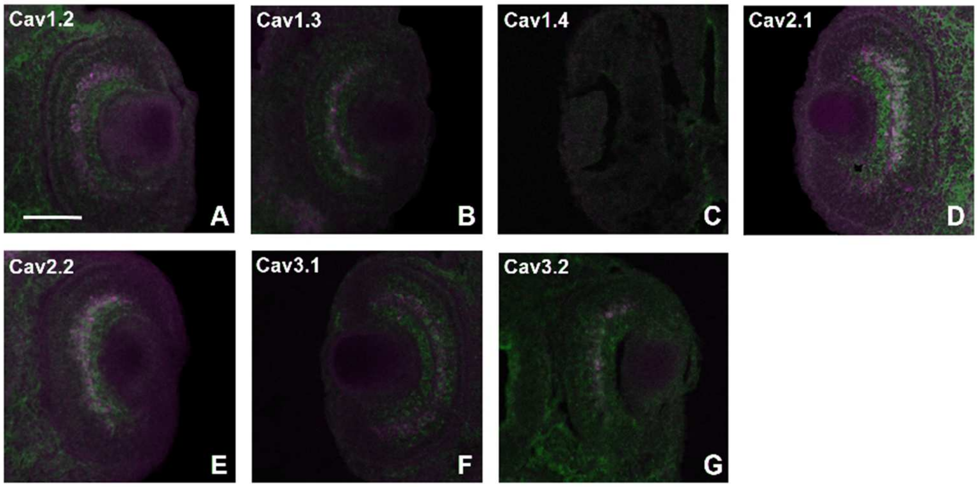

Supplementary Figure 4. (Magenta-green version of Figure 4 for the assistance of color-blind readers.) Coexpression patterns of xVIAAT and VGCC α1 subunits in the retina of Xenopus laevis swimming tadpole embryos. VGCC subunit expression is labeled with fluorescein (green) and xVIAAT expression is labeled with Cy3 (red). Coexpression is indicated by the yellow overlap of both channels. Scale bar represents 250 μm. xVIAAT coexpression with (A) Cav1.2, (B) Cav1.3, (C) Cav1.4, (D) Cav2.1, (E) Cav2.2, (F) Cav3.1, (G) Cav3.2. A magenta-green copy of this figure is provided as Supplementary Figure 4.
244x123mm (96 x 96 DPI)

Review
